# Functional Investigation of a Neuronal Microcircuit in the CA1 Area of the Hippocampus Reveals Synaptic Dysfunction in Dravet Syndrome Mice

**DOI:** 10.3389/fnmol.2022.823640

**Published:** 2022-03-16

**Authors:** Yael Almog, Anat Mavashov, Marina Brusel, Moran Rubinstein

**Affiliations:** ^1^Goldschleger Eye Research Institute, Sackler Faculty of Medicine, Tel Aviv University, Tel Aviv, Israel; ^2^The Department of Human Molecular Genetics and Biochemistry, Sackler Faculty of Medicine, Tel Aviv University, Tel Aviv, Israel; ^3^Sagol School of Neuroscience, Tel Aviv University, Tel Aviv, Israel

**Keywords:** Dravet syndrome, CA1 microcircuit, Nav1.1 voltage gated sodium channel, stratum-oriens, pyramidal neurons, Hm1a

## Abstract

Dravet syndrome is severe childhood-onset epilepsy, caused by loss of function mutations in the *SCN1A* gene, encoding for the voltage-gated sodium channel Na_V_1.1. The leading hypothesis is that Dravet is caused by selective reduction in the excitability of inhibitory neurons, due to hampered activity of Na_V_1.1 channels in these cells. However, these initial neuronal changes can lead to further network alterations. Here, focusing on the CA1 microcircuit in hippocampal brain slices of Dravet syndrome (DS, *Scn1a*^A1783V/WT^) and wild-type (WT) mice, we examined the functional response to the application of Hm1a, a specific Na_V_1.1 activator, in CA1 stratum-oriens (SO) interneurons and CA1 pyramidal excitatory neurons. DS SO interneurons demonstrated reduced firing and depolarized threshold for action potential (AP), indicating impaired activity. Nevertheless, Hm1a induced a similar AP threshold hyperpolarization in WT and DS interneurons. Conversely, a smaller effect of Hm1a was observed in CA1 pyramidal neurons of DS mice. In these excitatory cells, Hm1a application resulted in WT-specific AP threshold hyperpolarization and increased firing probability, with no effect on DS neurons. Additionally, when the firing of SO interneurons was triggered by CA3 stimulation and relayed via activation of CA1 excitatory neurons, the firing probability was similar in WT and DS interneurons, also featuring a comparable increase in the firing probability following Hm1a application. Interestingly, a similar functional response to Hm1a was observed in a second DS mouse model, harboring the nonsense *Scn1a*^R613X^ mutation. Furthermore, we show homeostatic synaptic alterations in both CA1 pyramidal neurons and SO interneurons, consistent with reduced excitation and inhibition onto CA1 pyramidal neurons and increased release probability in the CA1-SO synapse. Together, these results suggest global neuronal alterations within the CA1 microcircuit extending beyond the direct impact of Na_V_1.1 dysfunction.

## Introduction

Dravet syndrome (Dravet) is a developmental epileptic encephalopathy (DEE) of early childhood with an ominous course. Children develop normally during the first year of life, but subsequently exhibit febrile seizures that progress to prolonged spontaneous seizures, frequent episodes of status epilepticus, global developmental delay, and a high risk of sudden death (Dravet, 2011; Dravet and Oguni, 2013). The underlying genetic cause of Dravet, in ∼90% of the patients, is heterozygous loss of function mutations in the *SCN1A* gene, encoding for the alpha subunit of the voltage-gated sodium channel (Na_V_), Na_V_1.1 (Claes et al., 2001). Na_V_1.1 channels are crucial for neuronal excitability, contributing to the initiation and propagation of action potentials as well as amplification of synaptic depolarizations (Catterall et al., 2010). According to the prevailing hypothesis, the severity of *SCN1A*-related epileptic phenotypes correlates with the level of Na_V_1.1 loss of function. While haploinsufficiency in Na_V_1.1 activity results in Dravet, milder *SCN1A* mutations lead to less severe and treatable epilepsies, such as Generalized Epilepsy with Febrile Seizures Plus (GEFS+) and benign febrile seizures (Catterall et al., 2010; Nissenkorn et al., 2019).

Dravet mouse models (DS) are among the most accurate animal model representations of any human disease. Like Dravet patients, DS mice are mostly asymptomatic until their fourth week of life [postnatal day (P) 20–27], when they start experiencing seizures and profound premature mortality (Yu et al., 2006; Ogiwara et al., 2007, 2013; Cheah et al., 2012; Miller et al., 2014; Tsai et al., 2015; Mantegazza and Broccoli, 2019; Ricobaraza et al., 2019; Dyment et al., 2020; Fadila et al., 2020; Almog et al., 2021).

Studies of DS models that focused on recordings of single cortical or hippocampal neurons, in response to depolarizing current injections directly into the soma, demonstrated hypo-excitation of multiple types of inhibitory neurons, indicating that disinhibition serves as the root cause of Dravet (Yu et al., 2006; Kalume et al., 2007; Ogiwara et al., 2007; Mistry et al., 2014; Tai et al., 2014; Rubinstein et al., 2015b; De Stasi et al., 2016; Favero et al., 2018; Richards et al., 2018; Goff and Goldberg, 2019; Kuo et al., 2019; Dyment et al., 2020; Almog et al., 2021). Nevertheless, other studies proposed that the neuronal mechanism of Dravet is more complex. Specifically, changes in the activity of excitatory neurons, revealing reduced or enhanced activity, were reported (Ogiwara et al., 2013; Mistry et al., 2014; Salgueiro-Pereira et al., 2019; Almog et al., 2021; Studtmann et al., 2021). Moreover, the examination of spontaneous neuronal activity *in vivo* did not provide evidence for the predicted disinhibition (De Stasi et al., 2016; Tran et al., 2020). However, reduced inhibition can trigger multiple additional homeostatic network changes to stabilize neuronal firing (Antoine et al., 2019).

**FIGURE 1:**
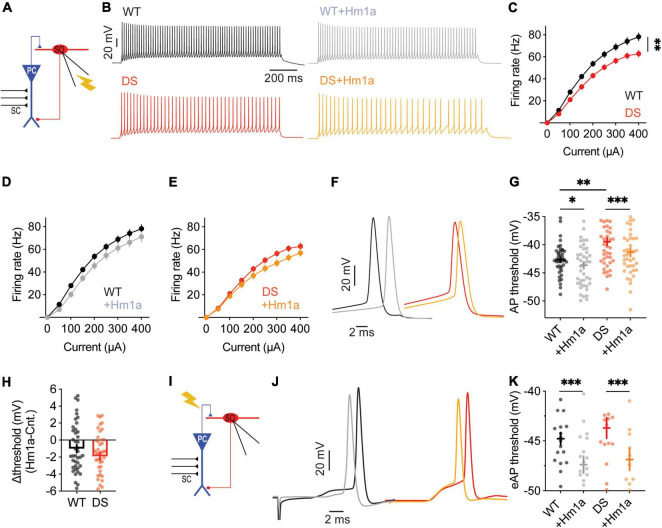
Stratum-oriens interneurons from WT and DS *Scn1a*^A1783V/WT^ mice respond similarly to Hm1a. **(A)** Illustration of the recording configuration. Firing properties of SO interneurons (red) were measured in response to current injection through the patch pipette **(B–H)**. **(B)** Representative traces of whole-cell current clamp recordings from WT and DS SO interneurons in response to current injection of +150 pA, before and after Hm1a application. **(C)** Firing frequency in response to depolarizing current injection at the indicated intensities in WT and DS. Statistical analysis utilized Mixed Model Repeated Measures ANOVA. **(D,E)** The effect of Hm1a on firing frequencies, in response to current injection through the whole-cell patch electrode in WT **(D)** and DS **(E)** mice. Statistical analysis utilized Two Way Repeated Measures ANOVA; **(D)** *p* = 0.36 for Hm1a treatment, *p* = 0.96 for current × treatment; **(E)** *p* = 0.37 for Hm1a treatment, *p* = 0.44 for current × treatment. **(F)** Representative WT and DS APs at rheobase, before and after Hm1a application. **(G)** The effect of Hm1a on AP threshold at rheobase current. Statistical analysis utilized Two Way Repeated Measures ANOVA. The markings on the graph depict the results of Bonferroni *post hoc* analysis: *p* = 0.001 (^**^) for genotype; *p* < 0.001 (^***^) for Hm1a treatment; *p* = 0.23 for the interaction. **(H)** The difference in AP threshold. The data in **(C–H)** included WT: *n* = 42 cells from 17 mice; DS: *n* = 43 cells from 14 mice. **(I)** Illustration of the recording configuration. Synaptic activation of SO interneurons (red) was triggered by antidromic activation of CA1 pyramidal neurons via stimulation of the alveus.**(J)** Representative traces of eAPs before and after Hm1a application. **(K)** The difference in eAP threshold. Statistical analysis utilized Two Way Repeated Measures ANOVA. The markings on the graph depict the results of Bonferroni *post hoc* analysis: *p* = 0.18 for genotype; *p* < 0.001 (^***^) for Hm1a treatment; *p* = 0.28 for the interaction. WT: *n* = 18 cells from 5 mice; DS: *n* = 15 cells from 4 mice.

Dysfunction of the hippocampal CA1 microcircuit was implicated before in Dravet (Liautard et al., 2013; Rubinstein et al., 2015b; Cheah et al., 2019; Stein et al., 2019; Almog et al., 2021). In this circuit, inputs from CA3 Schaffer collaterals (SC) activate CA1 pyramidal neurons. Next, recurrent collaterals of CA1 pyramidal cells activate stratum-oriens (SO)-residing horizontal interneurons, which in turn provide strong feedback inhibition to the same CA1 excitatory neurons (Figure 1A). This results in the modulation of the final output to extra-hippocampal regions (Müller and Remy, 2014).

Using acute brain slices from WT and DS mice, we studied the neuronal changes in both excitatory and inhibitory neurons within this microcircuit, focusing on the severe stage of epilepsy (P20–25). Previously, we demonstrated that CA1 SO interneurons are hypo-excitable in Dravet (Rubinstein et al., 2015b; Almog et al., 2021). However, the direct contribution of Na_V_1.1 loss of function to this phenotype was not tested. Moreover, alterations in synaptic communication within this circuit were not examined. The functional contribution of Na_V_1.1 was evaluated by measuring the functional response to the Na_V_1.1 activator, Hm1a (Osteen et al., 2016; Richards et al., 2018). Surprisingly, Hm1a had a similar effect on SO interneurons from WT and DS mice. Conversely, Hm1a had a smaller functional effect on DS CA1 pyramidal neurons. Additionally, to test the activity of the CA1 microcircuit, we examined the response of SO interneurons to SC stimulation, demonstrating similar firing probability in WT and DS, along with homeostatic synaptic changes within this circuit. Together, our results reveal multiple DS-related neuronal alterations, indicating that Dravet pathophysiology is highly complex, involving synaptic and neuronal function variations occurring concomitantly in excitatory and inhibitory neurons of the CA1 microcircuit.

## Materials and Methods

### Mice

All animal experiments were in accordance with the Animal Care and Use Committee (IACUC) of Tel Aviv University. Mice were group-housed in a standard animal facility at the Goldschleger Eye Institute at a constant (22°C) temperature, on a 12-h light/dark cycle, with *ad libitum* access to food and water.

Dravet syndrome mice harboring the heterozygous global *Scn1a*^A1783V/WT^ mutation were generated as reported before (Almog et al., 2021); briefly, males carrying the conditional floxed stop *Scn1a*^A1783Vfl^ allele [B6(Cg)-Scn1atm1.1Dsf/J; strain 026133; The Jackson Laboratory, Bar Harbor, ME, United States] were crossed with CMV-Cre females [B6⋅C-Tg(CMV-Cre)1Cgn/J; strain 006054; The Jackson Laboratory, Bar Harbor, ME, United States]. Both lines were maintained on the pure C57BL/6J genetic background. Both males and females, at similar proportions, were used for the electrophysiological recordings. DS *Scn1a*^A1783V/WT^ mice experience spontaneous convulsive seizures starting at P19, and all the mice experience such spontaneous seizures at the severe stage of the disease (Fadila et al., 2020; Almog et al., 2021). While the mice used for this study were not placed under video surveillance from P19 until the recording day, they displayed a characteristic DS behavior, including apparent hyperactivity and increased anxiety, and were easily distinguished from their WT littermates in their home cage. Based on these phenotypes, we speculate that these mice have experienced spontaneous convulsive seizures. Nevertheless, the genotype was still confirmed by genotyping after the experiment.

Dravet syndrome mice harboring the heterozygous nonsense *Scn1a*^R613X/WT^ (129S1/SvImJ-Scn1aem1Dsf/J; strain 034129, The Jackson Laboratory, Bar Harbor, ME, United States), were generated by crossing DS *Scn1a*^R613X/WT^ on the pure 129S1/SvImJ background with WT C57BL/6J mice (strain 000664), to generate mice on the mixed 50:50 129S1/SvImJ:C57BL/6J background. Both males and females were used for real-time PCR and electrophysiological recordings. While some mice were observed to have spontaneous seizures, they could not be distinguished from their WT littermates without genotyping.

### Brain Slice Electrophysiology

Hippocampal slices (300–400 μm thick) were prepared from WT or DS mice aged P20-25 as described previously (Rubinstein et al., 2015b; Almog et al., 2021), with minor modifications. Briefly, mice were decapitated under isoflurane anesthesia. The brain was quickly removed and placed into ice-cold slicing solution containing 75 mM sucrose, 87 mM NaCl, 25 mM NaHCO_3_, 25 mM D-glucose, 2.5 mM KCl, 1.25 mM NaH_2_PO_4_, 0.5 mM CaCl_2_ and 7 mM MgCl_2_. Slicing was performed using a Leica VT1200S vibratome (Leica Biosystems, Wetzlar, Germany). Parasagittal slices were used for whole-cell recordings of horizontal SO interneurons to better preserve the synaptic connections. The brain was cut midline and the hemispheres were glued on their callosal side onto a slope of 45°, forming longitudinal sagittal slices. To record CA1 pyramidal cells, the brain was glued on its ventral side, without any tilt, and horizontal slices were taken. Slices were transferred to a storage chamber with fresh artificial cerebrospinal fluid (ACSF) containing 125 mM NaCl, 3 mM KCl, 2 mM MgCl_2_, 2 mM CaCl_2_, 1.25 mM NaH_2_PO_4_, 26 mM NaHCO_3_, and 10 mM D-glucose, and incubated for 45 min at 37°C, followed by a 30 min recovery at room temperature. All solutions were saturated with 95% O_2_ and 5% CO_2_. The cells were visualized under oblique illumination with near-infrared LED and an upright microscope (SOM; Sutter Instrument, Novato, CA, United States). Horizontal SO interneurons were identified based on their horizontal fusiform somata and characteristic electrophysiological properties (Tricoire et al., 2011). Their characteristic morphology was also verified using biocytin (0.2%). Pyramidal cells were identified based on their location, morphology, and electrophysiological properties. Na_V_1.1 functional contribution was examined by application of 50 nM Hm1a (Alomone Labs, Jerusalem, Israel, Catalog #STH-601). Control experiments with vehicle, instead of Hm1a, confirmed the lack of effect on AP threshold (–46.2 ± 0.6 vs. –46.8 ± 0.4, *p* = 0.66), or firing probability (0.5 ± 0.1 vs. 0.6 ± 0.2, *p* = 0.5, *n* = 3).

Recordings were obtained using a Multiclamp 700B amplifier (Molecular Devices, San Jose, CA, United States) and Clampex 10.7 software (Molecular Devices, San Jose, CA, United States). For whole-cell current clamp recordings and measurements of paired pulse ratio (PPR), the patch pipette was filled with an internal solution containing: 145 mM K-Gluconate, 2 mM MgCl_2_, 0.5 mM EGTA, 2 mM ATP-Tris, 0.2 mM Na_2_-GTP and 10 mM HEPES, pH 7.2. To measure evoked EPSCs and IPSCs, we used an internal solution containing: 140 mM Cs-methanesulfonate, 5 mM CsCl, 2 mM MgCl_2_, 2 mM 2 ATP-Tris, 0.2 mM Na_2_-GTP, 10 mM HEPES and 5 mM QX-314. When filled with an intracellular solution, the patch electrode resistance ranged from 4 to 7 MΩ. Access resistance was monitored continuously for each cell. Only cells with access resistance lower than 30 MΩ, that were stable through the recording (changing by less than 20%) were included. The resting membrane potential was set to –60 mV by injection of no more than 50 pA, ranging from –50 to +50 pA. Bridge balance and pipette capacitance neutralization, based on the amplifier circuit, were applied to all current clamp recordings.

We used different paradigm for current clamp recordings, as illustrated in the Figures. Firing rates were determined by injection of 1 s long depolarizing current through the patch pipette. AP threshold was measured by injection of 10 ms long depolarizing current at rheobase, as we did before (Almog et al., 2021). To record synaptic evoked AP (eAP) from SO interneurons we stimulated the alveus; to evoke firing of pyramidal neurons, we stimulated the SC, as we did before (Almog et al., 2021). The stimulation strength was set to produce firing probability of 50% (i.e., 5 APs out of 10 stimuli at 1 Hz). Cells that produced 4 to 6 APs were used for analysis. For simultaneous field potential and whole-cell recordings (Figures 3–5), the stimulating electrode was put on the SC, the field electrode (1–2 MΩ, filled with ACSF) was placed on the stratum radiatum, 200–600 μm from the stimulating electrode; while patching the horizontal SO interneuron. The slopes of evoked extracellular field EPSPs (fEPSPs) were measured by calculating the linear regression of the initial part (20–80%) of the rising phase. The firing probability was calculated as the number of eAPs out of these 10 stimulations. For each cell, we recorded the response to different stimulus intensities. First, we determined the stimulation that produced firing of 50%, i.e., 5 APs out of 10 stimuli (1X). Next, we repeated the recording with half of the intensity (0.5X), as well as higher intensity (1.5X). We than repeated this recording after the addition of Hm1a (50 nM), without altering the stimulation strength, to look for changes in the firing probability. This stage was followed by the readjustment of the stimulation strength to reproduce a firing probability of 50%, which was used for measurements of the eAP threshold. The slice was changed following Hm1a application (to avoid any chances for partial wash off) and the perfusion pipes and chamber were washed thoroughly, for least 10 min, with ACSF prior to placing the next slice.

Paired-pulse ratio experiments were recorded in voltage clamp mode at a holding potential of –60 mV with an inter-stimulus interval (ISI) of 50 ms (Figures 6B–D, 7B–D). For EPSP measurements, we first determined the stimulation intensity that produced firing probability of 50%, then reset the intensity to half of that, and measured subthreshold responses to a train of stimulations given at 30 Hz. Neurons that fired eAP within this train of stimuli were excluded from the analysis (Figures 6E–G, 7E–G). Recordings of evoked EPSCs and IPSCs and excitation-inhibition balance were performed as described before (Antoine et al., 2019), with some adaptations. Briefly, membrane voltage was held at –60 or 0 mV for EPSC or IPSC recordings, respectively. The minimal response in Figures 6H–J, 7H–J was defined experimentally, as the stimulation intensity that produced a measurable current response in the recorded postsynaptic cell (1xEθ). As such, 1xEθ responses had a similar amplitude in both WT and DS. Next, EPSCs and IPSCs were measured in response to stronger stimuli (1.25–2.5xEθ). As the inhibitory neurons are not stimulated directly, IPSCs are the result of feed-forward and feedback inhibition.

Data were analyzed using Clampfit 10.7 (Molecular Devices), Igor Pro (WaveMetrics, Lake Oswego, OR, United States) and GraphPad Prism (GraphPad Software, La Jolla, CA, United States). AP threshold was defined as the voltage at which the first derivative of the AP waveform (dV/dt) reached 10 mV/ms.

### Real-Time PCR

Total RNA was extracted from the hippocampi of WT and DS *Scn1a*^R613X/WT^ mice at P20–25, using Purelink RNA mini kit according to the manufacturer’s instructions. cDNA was synthesized from 500 ng RNA using Maxima H Minus cDNA synthesis kit (Thermo Fisher Scientific, Life Technologies, Carlsbad, CA, United States). Real-time PCR reactions were performed in triplicates in a final volume of 10 μl with 5 ng of RNA as template, with TaqMan gene expression assays: *Scn1a* (Mm00450580_m1) and *Tfrc* (Mm00441941_m1) as endogenous control, on StepOnePlus real-time PCR system (Applied Biosystems, Thermo Fisher Scientific, Life Technologies, Carlsbad, CA, United States). Efficiency of 100%, dynamic range, and lack of genomic DNA amplification were verified. The relative expression was calculated as 2^–ΔΔCT^.

### Experimental Design and Statistical Analysis

Statistical analysis was performed using GraphPad Prism 9.2 (GraphPad Software, La Jolla, CA, United States). Two Way Repeated Measures ANOVA with Bonferroni *post hoc* analysis was used when multiple measurements were obtained from the same cell. Mixed Model Repeated Measures ANOVA was used to compare between firing rates in response to injection of 1 s long depolarizing current, and the excitation-inhibition balance. The Mann–Whitney test was used to compare two groups. The Correlations between fiber volley and fEPSP utilized linear regression analysis followed by statistical comparison of the slopes. The specific statistic comparison for each panel is indicated in the Figure legend. We defined *p* < 0.05 as statistically significant and marked *p < 0.05, ^**^p < 0.01, ^***^p < 0.001.

## Results

### Similar Response of Wild-Type and Dravet Syndrome *Scn1a*^A1783V/WT^ Stratum-Oriens Interneurons to Hm1a

Previous studies demonstrated a reduction of ∼50% in sodium currents of DS interneurons (Yu et al., 2006; Kalume et al., 2007; Mistry et al., 2014; Rubinstein et al., 2015b), as well as hypo-excitability of multiple types of GABAergic neurons. Similarly, the Dravet causing *Scn1a*^*A*1783*V*^ mutation was demonstrated to cause loss of function of Na_V_1.1 (Layer et al., 2021), as well as reduced firing and increased threshold for action potentials (APs) of inhibitory neurons (Kuo et al., 2019; Almog et al., 2021; Layer et al., 2021). These data indicate that reduced activity of Na_V_1.1 in inhibitory neurons is the cause for their reduced excitability. To directly test that here, we examined the change in the activity of SO interneurons before and after the application of Hm1a, a specific Na_V_1.1 activator. Hm1a inhibits the inactivation of Na_V_1.1, with a reduced effect on Na_V_1.3 and low impact on Na_V_1.2 and Na_V_1.6 (Osteen et al., 2016; Richards et al., 2018). We hypothesized a smaller effect of Hm1a on SO interneurons from DS mice, in accordance with Na_V_1.1 haploinsufficiency.

First, we confirmed that DS *Scn1a*^A1783V/WT^ SO interneurons were hypo-excitable (Figures 1B,C), similar to previous data (Rubinstein et al., 2015b; Almog et al., 2021). Moreover, an examination of the threshold for AP demonstrated a more depolarized threshold voltage in DS (Figure 1G), in accordance with reduced firing of these SO interneurons. Next, we examined the effect of the application of Hm1a, at 50 nM, a concentration that was previously found to enhance the firing of fast spiking interneurons in DS mice (Richards et al., 2018; Goff and Goldberg, 2019; Chever et al., 2021). The application of Hm1a had no significant effect on the firing rates of WT and DS (Figures 1D,E). While this was somewhat unexpected, lack of effect on the firing frequency was reported before by Richards et al. (2018), who reported that Hm1a did not increase the firing rates of non-collapsing WT CA1 GABAergic interneurons. Nevertheless, Hm1a hyperpolarized the AP threshold in both WT and DS *Scn1a*^A1783V/WT^ (Figures 1F,G). Intriguingly, the extent of this Hm1a-induced threshold shift showed a slightly smaller effect in WT, resulting in the dissipation of the difference between the threshold levels of WT and DS following Hm1a application (Figures 1F–H).

Under physiological synaptic activation, excitatory inputs are received by the dendrites, integrated and transferred through the cell soma to the axon initial segment (AIS) (Stuart and Spruston, 2015). Conversely, injection of prolonged depolarizing current, close to the AIS, bypasses this natural path, resulting in direct AIS depolarization, that may lead to misinterpretation of the data. Therefore, to test the effect of Hm1a following synaptically evoked firing, we stimulated the axons of CA1 pyramidal neurons, located in the alveus, while recording the postsynaptic response of SO interneurons under whole-cell current clamp (Figure 1I). The stimulation strength was set to produce firing probability of 50%, representing the middle of their dynamic range of response, resulting in 5 synaptically evoked APs (eAPs) out of 10 stimuli that were given at 1 Hz, as we did before (Rubinstein et al., 2015b; Almog et al., 2021). However, the effect of Hm1a was similar in both genotypes, again, with hyperpolarization of the threshold by ∼3 mV (Figures 1J,K). Together, these data show that while SO interneurons are hypo-excitable in DS *Scn1a*^A1783V/WT^, demonstrated by reduced firing and increased threshold, the functional change in response to Hm1a is comparable between WT and DS.

### CA1 Pyramidal Neurons From Dravet Syndrome *Scn1a*^A1783V/WT^ Mice Demonstrate Reduced Response to Hm1a

While Na_V_1.1 channels were shown to be expressed in pyramidal excitatory neurons (Westenbroek et al., 1989; Yu et al., 2006; Cembrowski et al., 2016) and several studies demonstrated their structural and functional changes in DS (Mistry et al., 2014; Tsai et al., 2015; Salgueiro-Pereira et al., 2019; Almog et al., 2021), others reported unaltered excitability of these cells (Yu et al., 2006; Rubinstein et al., 2015b; De Stasi et al., 2016; Favero et al., 2018). Here we tested the functional response of CA1 pyramidal cells to Hm1a.

**FIGURE 2:**
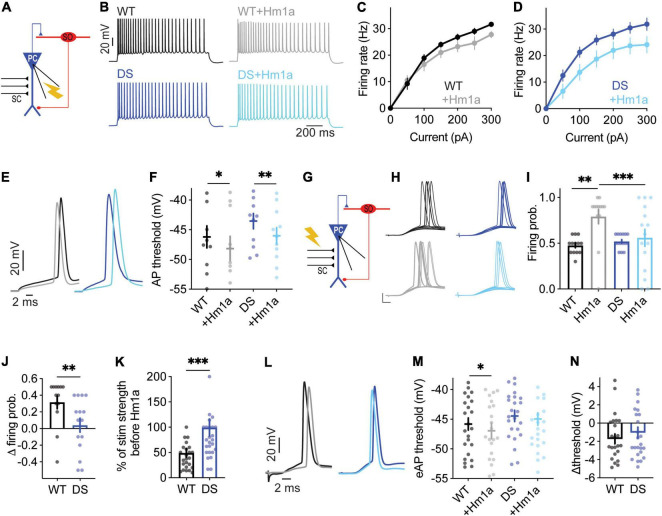
Reduced evoked synaptic response of DS *Scn1a*^A1783V/WT^ CA1 pyramidal neurons to Hm1a application. **(A)** Illustration of the recording configuration. Neuronal firing of CA1 pyramidal neurons (blue) was evoked by injection of depolarizing current through the patch pipette. **(B)** Representative traces of whole-cell current clamp recordings from CA1 pyramidal neurons in response to current injection of +150 pA, before and after Hm1a application. **(C,D)** The effect of Hm1a on firing in response to current injection through the whole-cell patch electrode in WT **(C)** and DS **(D)** CA1 pyramidal neurons. Statistical analysis utilized Mixed Model Analysis of Variance; **(C)** *p* = 0.08 for Hm1a treatment, *p* = 0.06 for current × treatment; **(D)** *p* = 0.07 for Hm1a treatment, *p* = 0.09 for current × treatment. **(E)** Representative WT and DS APs at rheobase, before and after Hm1a application. **(F)** The effect of Hm1a on AP threshold at rheobase current. Statistical analysis utilized Two Way Repeated Measures ANOVA. The markings on the graph depict the results of Bonferroni *post hoc* analysis: *p* = 0.30 for genotype; *p* < 0.001 (^***^) for Hm1a treatment, and *p* = 0.58 for the interaction. The data in **(C–F)** included: WT: *n* = 11 cells from 4 mice; DS: *n* = 16 cells from 3 mice. **(G)** Schematic illustration of the SC stimulation and whole-cell recording of a CA1 pyramidal neuron. **(H)** Representative traces of WT and DS CA1 pyramidal neurons in response to a train of 10 stimuli at 1 Hz, delivered to the SC, with or without Hm1a. Scale bar = 20 mV, 5 ms. **(I,J)** The effect of Hm1a on the firing probability of WT and DS CA1 pyramidal neurons **(I)**, and the difference in firing probability calculated for each cell **(J)**. Statistical analysis for the data presented in **I** utilized Two Way Repeated Measures ANOVA. The markings depict the results of Bonferroni *post hoc* analysis: *p* = 0.036 (*) for genotype; *p* = 0.070 for Hm1a treatment; *p* = 0.002 (^**^) for the interaction. The Mann–Whitney test was used to analyze the data in **(J)**. **(K)** The effect of Hm1a on the stimulation intensity required to produce 50% firing probability. Statistical analysis utilized the Mann–Whitney test. **(L–N)** Representative traces of eAPs before and after Hm1a application **(L)**, the overall effect of Hm1a on the eAP threshold **(M)**, and the difference in eAP threshold **(N)**. Statistical analysis utilized Two Way Repeated Measures ANOVA. The markings depict the results of Bonferroni *post hoc* analysis: *p* = 0.3 for genotype; *p* = 0.019 (*) for Hm1a treatment; *p* = 0.19 for the interaction. The data in **(I–N)** included WT: *n* = 25 cells from 9 mice; DS: *n* = 27 cells from 8 mice.

When firing was evoked by direct injections of depolarizing current through the patch pipette, the application of Hm1a had no significant effect on the firing rate in either WT or DS *Scn1a*^A1783V/WT^ (Figures 2A–D). However, the application of Hm1a resulted in AP threshold hyperpolarization, that was comparable in both genotypes (Figures 2E,F). Next, to examine the effect of Hm1a on synaptically evoked firing and eAP threshold, the stimulating electrode was placed on CA3 SC, and the stimulation intensity was set to produce firing probability of 50% in the recorded postsynaptic CA1 excitatory cell (Figure 2G). Under this experimental paradigm, the application of Hm1a increased the firing probability of WT CA1 pyramidal neurons (Figures 2H–J). Next, in the presence of Hm1a, we readjusted the stimulation strength to reproduce a firing probability of 50%. In WT CA1 pyramidal neurons, this required about half of the original stimulation strength, in accordance with the initial increased firing probability (Figure 2K). In addition, Hm1a application caused a small, but significant, hyperpolarization of the threshold for eAP (Figures 2L–N). Conversely, Hm1a had no significant effect on the firing probability or eAP threshold in CA1 pyramidal neurons of DS *Scn1a*^A1783V/WT^ mice (Figures 2H–N). Together, these data demonstrated reduced response of CA1 pyramidal neurons in DS mice to Hm1a.

### Dravet Syndrome *Scn1a*^A1783V/WT^ Mice Feature Reduced Synaptic Activation of CA1 Pyramidal Neurons and Preserved Firing of Stratum-Oriens Interneurons

**FIGURE 3:**
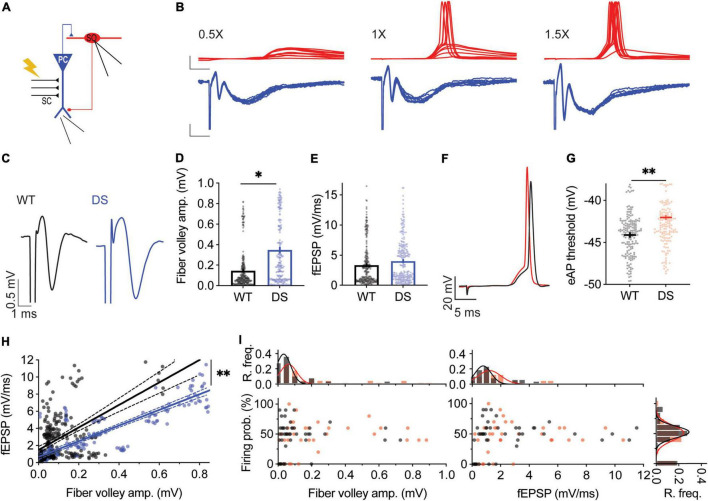
Preserved firing of DS *Scn1a*^A1783V/WT^ SO interneurons in response to CA3 stimulation. **(A)** Illustration of the experimental setup. The stimulating electrode was placed on the CA3, with extracellular field potentials recorded from CA1 pyramidal neurons (blue), concomitantly with whole-cell recordings of a SO interneuron (red). **(B)** Representative traces of field potential of CA1 pyramidal neurons (blue) and whole-cell recording of a SO interneuron (red) at different stimulation intensities. Left: low stimulation intensity (0.5X) that produced subthreshold responses in the recorded SO interneuron. Middle: intermediate stimulation intensity (1X) that produced firing probability of 50%, i.e., 5 AP and 5 EPSPs out of 10 stimulations at 1 Hz. Right: stronger stimulation intensity (1.5X), that produced firing probability of 90% in the recorded SO interneuron. Scale bar: top: 20 mV, 5 ms; bottom: 0.2 mV, 5 ms. **(C)** Representative traces of WT (black) and DS (blue) field potentials. **(D,E)** Average fiber volley amplitudes **(D)** and fEPSP **(E)** in recordings at which the firing probability of SO interneurons was 50% (1X). Statistical analysis utilized Mixed Model Analysis of Variance. **(F)** Representative traces of WT (black) and DS (red) synaptically evoked APs (eAPs) recorded from SO interneuron. **(G)** Average eAP threshold of WT and DS SO interneurons. Statistical analysis utilized Mixed Model Analysis of Variance. **(H)** Linear regression analysis of fEPSP slope vs. fiber volley amplitude. The solid lines represent the linear fit, and the dashed lines depict the 95% confidence interval. The fitted equation was *Y* = 13.14*X + 1.4 for WT (*R*^2^ = 0.33) and Y = 9.14*X + 0.67 in DS (*R*^2^ = 0.72). The statistical difference between the slopes is presented (*p* = 0.0052). We repeated this analysis by fitting a non-linear exponential model to the data. The goodness of the fit was: WT: *R*^2^ = 0.33, DS: *R*^2^ = 0.77, and the statistical difference between the curves was *p* < 0.001. **(I)** SO interneurons firing probability vs. fiber volley amplitude or fEPSP. R.freq: the relative frequency histograms and Gaussian fit (solid line) of fiber volley amplitude, fEPSP or SO firing probability in WT and DS. WT: 28 cells from 11 mice; DS: 23 cells from 7 mice.

With the differential effect of Hm1a on excitatory and inhibitory neurons, we set to simultaneously probe the activity of excitatory and inhibitory neurons within the CA1 microcircuit. We stimulated the CA3 SC while monitoring synaptic responses of CA1 pyramidal neurons using extracellular field-potential measurements, concomitantly with whole-cell recordings of SO interneurons (Figure 3A). This experimental paradigm enables the measurements of fiber volley amplitudes, reporting the level of CA3 stimulation, the field excitatory post-synaptic potential (fEPSP) initial slope, corresponding mostly to the synaptic responses of CA1 pyramidal neurons (Hawkins et al., 2016), and SO responses under whole-cell current clamp. For each SO cell, the CA3 SC were stimulated at different amplitudes (1X, 0.5X, and 1.5X) (Figure 3B). 1X stimulation was defined as the stimulation intensity that resulted in a firing probability of 50% in the recorded SO interneuron. Next, for the same cell, the stimulation intensity was reduced by half (0.5X) or increased by half (1.5X), to cover a wide dynamic range of responses. Low stimulation intensities of CA3 SC (0.5X) induced field responses in CA1 pyramidal neurons, with almost no eAPs in the recorded SO interneuron (Figure 3B, left). Conversely, stronger suprathreshold stimulation (1.5X) resulted in CA1 depolarization, followed by high firing probability of SO interneurons (Figure 3B, right). Focusing on stimulation intensities yielding a 50% SO firing probability (1X), larger fiber volley amplitudes, with similar fEPSP slopes were measured in DS *Scn1a*^A1783V/WT^ (Figures 3C–E). These results indicate that stronger SC stimulation is needed to produce comparable synaptic responses in DS CA1 pyramidal neurons. Analyses of the threshold for eAP of SO interneurons, using this experimental paradigm, demonstrated a more depolarized voltage in DS *Scn1a*^A1783V/WT^ (Figures 3F,G), in agreement with reduced excitability of these interneurons (Figures 1C,G). Analysis of the relationship between fiber volley amplitude and fEPSP, across the full range of responses (0.5X, 1X, 1.5X), revealed that for the same fiber volley amplitude, lower fEPSP was measured in DS *Scn1a*^A1783V/WT^ (Figure 3H). Together, these recordings suggest that in DS the CA3-CA1 synaptic strength is weaker, and the excitability of SO interneurons is reduced. Based on these measurements, we expected an additive reduction in the firing probability of SO interneurons, following SC stimulation. However, surprisingly, the firing probability of SO interneurons, along the range of fiber volley amplitudes or CA1 fEPSP tested, was similar between WT and DS (Figure 3I) indicating a preserved activity of SO interneurons under these conditions, in response to a low frequency (1 Hz) stimulation.

**FIGURE 4:**
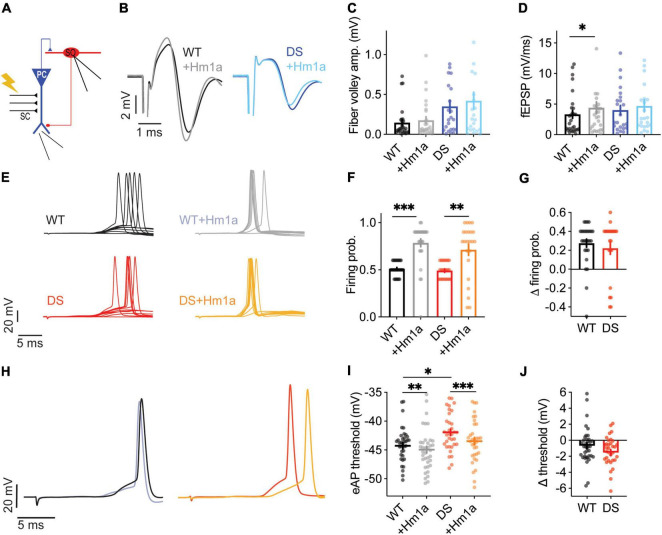
Hm1a increased the firing of DS *Scn1a*^A1783V/WT^ SO interneurons following SC stimulation. **(A)** Illustration of the experimental setup. **(B)** Representative traces of WT (black or gray) and DS (dark or pale blue) field potentials before and after Hm1a application. **(C,D)** Average fiber volley amplitudes **(C)** and fEPSP **(D)** before and after Hm1a application. Statistical analysis utilized Two Way Repeated Measures ANOVA. The markings depict the results of Bonferroni *post hoc* analysis. In **(C)** *p* = 0.02 (*) for genotype; *p* = 0.02 (*) for Hm1a treatment; *p* = 0.33 for the interaction. For **(D)** *p* = 0.66 for genotype; *p* = 0.01 (*) for Hm1a treatment; *p* = 0.54 for the interaction. The stimulation intensities in these experiments were 328 ± 84.6 mA in WT and 1045 ± 259.5 mA in DS (*p* = 0.002, Mann–Whitney test). **(E)** Representative traces of WT and DS SO interneurons in response to a train of 10 stimuli at 1 Hz, delivered to the SC, with or without Hm1a. **(F,G)** The effect of Hm1a on the firing probability of WT and DS SO interneurons **(F)**, and the difference in firing probability calculated for each cell **(G)**. Firing probability was calculated as the number of eAPs divided by 10 (stimuli). Statistical analysis for the data presented in F utilized Two Way Repeated Measures ANOVA. The markings depict the results of Bonferroni *post hoc* analysis: *p* = 0.22 for genotype; *p* < 0.0001 (^***^) for Hm1a treatment; *p* = 0.47 for the interaction. **(H)** Representative traces of eAPs before and after Hm1a application. **(I)** The effect of Hm1a on the eAP threshold. Statistical analysis utilized Two Way Repeated Measures ANOVA. The markings depict the results of Bonferroni *post hoc* analysis: *p* = 0.02 (*) for genotype; *p* < 0.0001 (^***^) for Hm1a treatment; *p* = 0.18 for the interaction. **(J)** The difference in eAP threshold. WT: *n* = 28 cells from 11 mice; DS: *n* = 23 cells from 7 mice.

The effect of Hm1a using this recording configuration was also tested (Figure 4A). Analysis of the extracellular field recordings of CA1 excitatory neurons demonstrated that Hm1a application did not change the fiber volley, but the fEPSP was increased in WT CA1 (Figures 4B–D). Conversely, in recordings of DS *Scn1a*^A1783V/WT^ CA1, Hm1a did not affect the fiber volley or fEPSP slope (Figures 4B–D). Thus, Hm1a-induced enhancement of synaptic responses in CA1 pyramidal neurons was not observed in DS *Scn1a*^A1783V/WT^.

Given that Hm1a did not increase the firing probability or the fEPSP of CA1 pyramidal cells from DS mice (Figures 2G–I, 4D), we expected an overall smaller increase in the firing probability of SO *Scn1a*^A1783V/WT^ interneurons, when their firing was initiated by stimulation of the CA3 SC and relayed by CA1 activation (Figure 4A). However, Hm1a increased the firing probability of SO interneurons in WT and DS *Scn1a*^A1783V/WT^ by ∼30% (Figures 4E–G). Interestingly, Hm1a also affected the spike timing in DS, causing slight delay, that was only observed in DS SO interneurons (WT: 18.45 ± 0.48 ms, vs. 18.99 ± 0.4 ms after Hm1a, Bonferroni *post hoc* analysis *p* = 0.15; DS: 15.58 ± 0.6 ms vs. 16.86 ± 0.6 ms after Hm1a, *p* = 0.006).

Moreover, despite the more depolarized eAP threshold in DS (Figures 3F,G), Hm1a similarly hyperpolarized the eAP threshold in WT and DS (Figures 4H–J). Thus, interestingly, despite the lower contribution of Hm1a-sensitive channels to CA1 field response and pyramidal neurons firing in DS *Scn1a*^A1783V/WT^, enhancement of the firing probability of SO interneurons was preserved, suggesting a possible compensatory mechanism within the CA1 microcircuit.

### Hm1a Increases the Firing Probability of Stratum-Oriens Interneurons From Dravet Syndrome Mice Harboring the Nonsense *Scn1a*^R613X/WT^ Mutation

As the preserved Hm1a effect in DS *Scn1a*^A1783V/WT^ was surprising, we repeated these experiments in another DS model, harboring the heterozygous nonsense *Scn1a*^R613X/WT^ mutation. Notably, these mice were kept on a mixed 129S1/SvImJ:C57BL/6J genetic background and therefore displayed milder epileptic phenotypes compared to DS *Scn1a*^A1783V/WT^ on the pure C57BL/6J background (Yu et al., 2006; Miller et al., 2014; Mistry et al., 2014; Rubinstein et al., 2015b).

**FIGURE 5:**
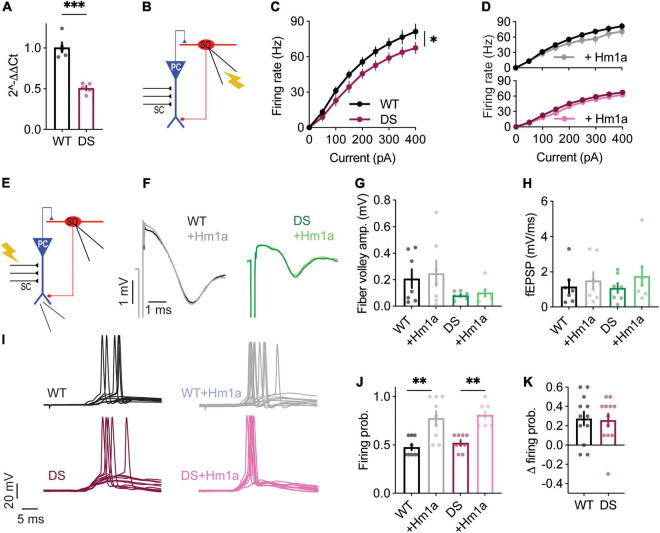
Hm1a increased the firing of DS *Scn1a*^R613X/WT^ SO interneurons following SC stimulation. **(A)** Quantitative real-time PCR analysis of hippocampal *Scn1a* mRNA expression, *n* = 5 in each genotype. Statistical analysis utilized unpaired *t*-test. **(B)** Illustration of the experimental setup used for **(C,D)**. **(C)** Firing frequency in response to depolarizing current injection at the indicated intensities in WT and DS. **(D)** The effect of Hm1a on firing frequencies, in response to current injection through the whole-cell patch electrode in WT and DS mice. Statistical analysis in panels **(C,D)** utilized Mixed Model Repeated Measures ANOVA. **(E)** Illustration of the experimental setup used for **(F–K)**. **(F)** Representative traces of WT (black or gray) and DS (green or pale green) field potentials before and after Hm1a application. **(G,H)** Average fiber volley amplitudes **(G)** and fEPSP **(H)** before and after Hm1a application. Statistical analysis utilized Two Way Repeated Measures ANOVA with Bonferroni *post hoc* analysis. In **(G)** *p* = 0.12 for genotype; *p* = 0.35 for Hm1a treatment; *p* = 0.76 for the interaction. For **(H)** *p* = 0.88 for genotype; *p* = 0.06 for Hm1a treatment; *p* = 0.51 for the interaction. The stimulation intensities in these experiments were 394 ± 68.7 mA in WT and 160 ± 48 mA in DS (*p* = 0.014, Mann–Whitney test). **(I)** Representative traces of WT and DS SO interneurons in response to a train of 10 stimuli at 1 Hz, delivered to the SC, with or without Hm1a. **(J,K)** The effect of Hm1a on the firing probability of WT and DS SO interneurons **(J)**, and the difference in firing probability calculated for each cell **(K)**. Firing probability was calculated as the number of eAPs divided by 10 (stimuli). Statistical analysis utilized Two Way Repeated Measures ANOVA. The markings depict the results of Bonferroni *post hoc* analysis: *p* = 0.28 for genotype; *p* < 0.0001 (^***^) for Hm1a treatment; *p* = 0.91 for the interaction. In these experiments Hm1a had a tendency towards hyperpolarization of the eAP threshold (WT: –46.19 ± 1 vs. –47.6 ± 1.4 after Hm1a; DS: –43.65 ± 1 vs. –46.1 ± 2.6 after Hm1a). WT: *n* = 9 cells from 4 mice; DS: *n* = 10 cells from 4 mice.

Quantitative real-time PCR analysis confirmed the expected reduction in *Scn1a* mRNA expression in the hippocampus (Figure 5A), as well as reduced firing rates of SO interneurons from *Scn1a*^R613X/WT^ DS mice in response to prolonged injection of depolarizing current (Figures 5B,C). Similar to our data with DS *Scn1a*^A1783V/WT^, Hm1a did not affect the firing rates of *Scn1a*^R613X/WT^ SO interneurons following prolonged current injections (Figure 5D).

Next, we tested the effect of Hm1a following stimulation of the CA3 SC, with concomitant CA1 field potential recordings and whole cell recordings of SO interneurons (Figure 5E). In these DS mice, Hm1a had no effect on synaptic responses of CA1 excitatory neurons (Figures 5F–H), and a tendency toward hyperpolarization of the eAP threshold, but with no statistical effect, in either genotype (WT: –46.19 ± 1 vs. –47.6 ± 1.4 after Hm1a; DS: –43.65 ± 1 vs. –46.1 ± 2.6 after Hm1a). Nevertheless, Hm1a application significantly increased the firing probability of SO interneurons in both WT and DS *Scn1a*^R613X/WT^ by ∼30% (Figures 5I–K), resembling the increase we observed in DS *Scn1a*^A1783V/WT^ (Figures 4E–G).

Genetic background greatly affects the severity of the epileptic phenotypes and the function of excitatory and inhibitory neurons (Mistry et al., 2014; Rubinstein et al., 2015b). Therefore, while additional studies are needed for thorough characterization of the epileptic phenotypes of DS *Scn1a*^R613X/WT^, variations in the extent of Hm1a effect on CA1 field response and eAP threshold may be related to the mixed 129S1/SvImJ:C57BL/6J background of these mice. Nevertheless, these data demonstrate that the application of Hm1a comparably increases the firing of SO interneurons from WT mice as well as DS mice harboring the nonsense *Scn1a*^R613X^ or missense *Scn1a*^*A*1783*V*^ mutation.

### Reduced Excitation and Inhibition Onto CA1 Pyramidal Neurons of Dravet Syndrome *Scn1a*^A1783V/WT^ Mice

**FIGURE 6:**
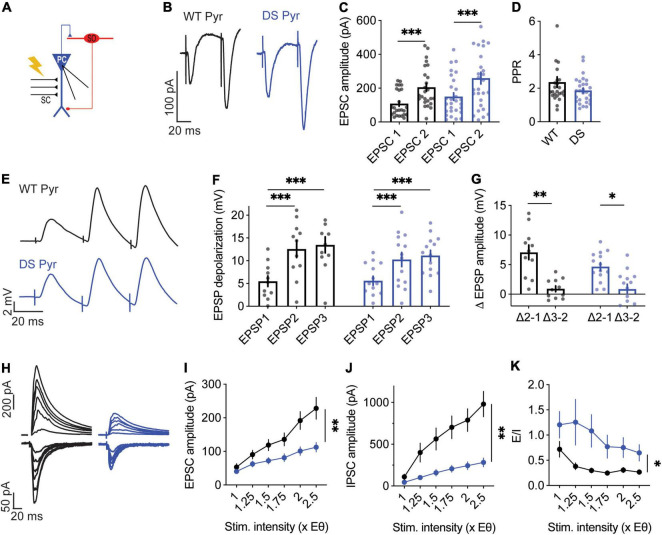
Altered excitatory and inhibitory synaptic currents in DS *Scn1a*^A1783V/WT^ CA1 pyramidal neurons. **(A)** Illustration of the stimulation setup. The stimulating electrode was placed on the CA3 SC while the CA1 pyramidal neurons were measured using whole-cell patch clamp. **(B)** Representative traces of WT (black) and DS (blue) paired-pulse responses. **(C)** Average EPSC amplitudes of the 1st and 2nd stimuli that were given within 50 ms. Statistical analysis utilized Two Way repeated measures ANOVA. The markings depict the results of Bonferroni *post hoc* analysis: *p* = 0.167 for genotype; *p* < 0.0001 (^***^) for the EPSC number; and *p* = 0.62 for the interaction. **(D)** Paired-pulse ratio (EPSP2/EPSP1). Statistical analysis utilized the Mann–Whitney test. WT: *n* = 13 cells from 4 mice, DS: *n* = 13 cells from 3 mice. **(E)** Representative EPSPs in WT and DS CA1 pyramidal neurons in response to a train of stimuli at 30 Hz. **(F)** EPSP amplitude at each of the three stimuli. Statistical analysis utilized Two Way Repeated Measures ANOVA. The markings depict the results of Bonferroni *post hoc* analysis: *p* = 0.43 for genotype; *p* < 0.001 (^***^) for the EPSP number; *p* = 0.07 for the interaction. **(G)** The added depolarization of each EPSP. Statistical analysis utilized Two Way Repeated Measures ANOVA. The markings depict the results of Bonferroni *post hoc* analysis: *p* = 0.052 for genotype; *p* < 0.001 (^***^) for the EPSP number; *p* = 0.25 for the interaction. WT: *n* = 13 cells from 4 mice; DS: *n* = 13 cells from 3 mice. **(H–J)** Representative traces **(H)**, average EPSCs **(I)** and IPSCs **(J)** evoked by SC stimulation at different stimulation intensities from WT and DS mice. EPSCs were measured at a holding potential –60 mV, and IPSCs were measured at 0 mV. Statistical analysis utilized Two Way Repeated Measures ANOVA. For the EPSCs: *p* = 0.009 (^**^) for genotype; *p* < 0.001 (^***^) for the interaction; for the IPSCs: *p* = 0.001 (^**^) for genotype; *p* < 0.001 (^***^) for the interaction. **(K)** E/I ratio. Statistical analysis utilized Mixed Model Repeated Measures ANOVA: *p* = 0.01 (*) for genotype; *p* = 0.001 (^**^) for the stimulation intensity; *p* = 0.3 for the interaction. WT: *n* = 15 cells from 3 mice; DS: *n* = 13 cells from 3 mice.

Higher fiber volley amplitudes were observed in DS *Scn1a*^A1783V/WT^, while the fEPSP was similar in both genotypes (Figures 3C–E,H). These properties may result from alterations in excitatory synaptic transmission within the CA3-CA1 axis by reduced neurotransmitter release from CA3 terminals, inhibited CA1 pyramidal neurons postsynaptic response, or changes in the ratio between excitation and inhibition. To further examine the properties of CA3 release, we used the paired pulse ratio (PPR) protocol (Zucker and Regehr, 2002). For each postsynaptic CA1 neuron, we first measured the stimulation strength needed to produce firing probability of 50% (1X, under current clamp), and used half of this stimulation (0.5X) to record the PPR responses. The EPSCs and PPR were similar between WT and DS mice, suggesting similar presynaptic properties (Figures 6A–D). Next, to examine the processing of synaptic excitation by CA1 neurons, we switched to current-clamp mode and measured the change in voltage in response to SC stimulation at 30 Hz. These recordings also demonstrated comparable postsynaptic CA1 responses, with an overall similar depolarization with each stimulus in both WT and DS neurons (Figures 6E–G), suggesting similar processing of excitatory inputs.

In addition, we examined the properties of EPSCs and inhibitory post-synaptic currents (IPSCs), and the balance between them. In this protocol, we measured the minimal stimulation required to evoke a measurable response (1xEθ, EPSCs or IPSCs), followed by increasing the stimulation intensity up to 2.5-fold of the initial minimal intensity (1–2.5×Eθ). Notably, in this protocol stimulation intensity is normalized to the minimal response, in contrast to the measurements of PPR or synaptic depolarization, in which the stimulation was stronger and normalized to suprathreshold stimulation, leading to the firing probability of 50%. Therefore, these different stimulation regimens enable to examine a wide spectrum of synaptic stimulations. As expected, the amplitude of the minimal response was similar between DS *Scn1a*^A1783V/WT^ and WT. Conversely, with increasing stimulation strength, the EPSCs and IPSCs amplitudes were lower in DS (Figures 6H–J), indicating reduced synaptic conductance in DS. The ratio between excitation and inhibition was shown to be increased in DS (Han et al., 2012; Rubinstein et al., 2015a). In accordance, the calculation of the ratio between excitatory and inhibitory currents (E/I balance), demonstrated enhanced excitation in DS, indicating that despite an overall reduction in synaptic conductances, the inhibition was further reduced (Figure 6K). Together, our data demonstrate reduced synaptic currents in the CA3-CA1 synapse. While reduced synaptic excitation correlates with larger fiber volley amplitudes measured in DS *Scn1a*^A1783V/WT^ CA1 neurons (Figures 3C–E,H), increased E/I balance is in line with previous studies of DS mice (Mantegazza and Broccoli, 2019).

### Reduced Facilitation in the CA1-SO Synapse in Dravet Syndrome *Scn1a*^A1783V/WT^ Mice

**FIGURE 7:**
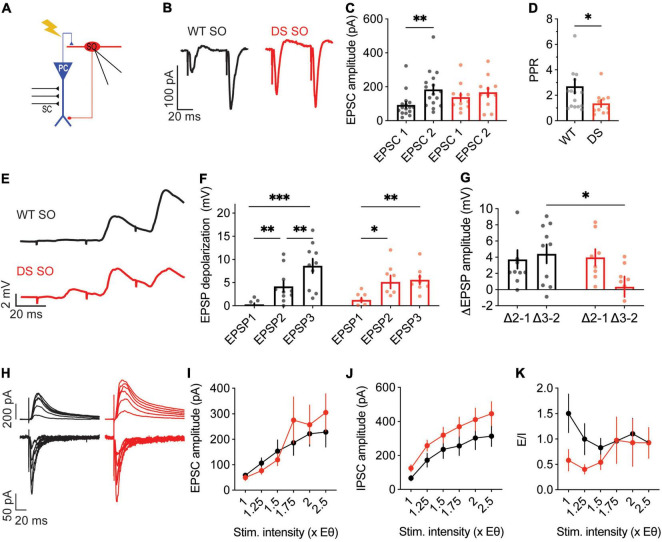
Reduced paired pulse facilitation, with normal excitatory and inhibitory synaptic currents, in DS *Scn1a*^A1783V/WT^ SO interneurons. **(A)** Illustration of the experimental setup. Synaptic activation of SO interneurons was triggered by stimulation of the alveus. **(B)** Representative traces of WT (black) and DS (red) paired-pulse responses. **(C)** Average EPSC amplitudes of the 1st and 2nd stimuli that were given within 50 ms. Statistical analysis utilized Two Way Repeated Measures ANOVA. The markings depict the results of Bonferroni *post hoc* analysis: *p* = 0.62 for genotype, *p* = 0.01 (*) for the EPSC number, *p* = 0.16 for the interaction. **(D)** Paired-pulse ratio (EPSP2/EPSP1). Statistical analysis utilized the Mann–Whitney test. WT: *n* = 17 cells from 5 mice; DS: *n* = 11 cells from 4 mice. **(E)** Representative EPSPs in WT and DS SO interneurons in response to a train of stimuli at 30 Hz. **(F)** EPSP amplitude at each of the three stimuli. Statistical analysis utilized Two Way Repeated Measures ANOVA. The markings depict the results of Bonferroni *post hoc* analysis: *p* = 0.75 for genotype; *p* < 0.001 (^***^) for the EPSP number, *p* = 0.052 for the interaction. **(G)** The added depolarization of each EPSPs. Statistical analysis utilized Two Way Repeated Measures ANOVA. The markings depict the results of Bonferroni *post hoc* analysis: *p* = 0.07 for genotype; *p* = 0.28 for the EPSP added depolarization; *p* = 0.12 for the interaction. WT: *n* = 10 cells from 6 mice; DS: *n* = 9 cell from 5 mice. **(H–J)** Representative traces **(H)** and average EPSCs **(I)** and IPSCs **(J)** evoked by alveus stimulation at different stimulation intensities from WT and DS mice. EPSCs were measured at a holding potential –60 mV, and IPSCs were measured at 0 mV. Statistical analysis utilized Two Way Repeated Measures ANOVA. For the EPSCs: *p* = 0.7 for genotype; *p* = 0.4 for the interaction; for the IPSCs: *p* = 0.15 for genotype; *p* = 0.68 for the interaction. **(K)** E/I ratio. Statistical analysis utilized Mixed Model Repeated Measures ANOVA: *p* = 0.1 for genotype; *p* = 0.3 for the stimulation intensity; *p* = 0.51 for the interaction. WT: *n* = 12 cells from 3 mice; DS: *n* = 11 cells from 3 mice.

Similar firing of WT and DS SO interneurons, under CA3 stimulation, may also stem from altered synaptic communication in the CA1-SO axis, such as changes in release probability, processing of synaptic depolarization or E/I balance. First, we examined the PPR of the CA1-SO synapse. The stimulation strength was set to 0.5X, as we did in the CA3-CA1 analysis. Examination of the PPR demonstrated greater facilitation in WT compared to DS *Scn1a*^A1783V/WT^ SO interneurons, indicating higher release probability in the first pulse in DS (Figures 7A–D). Next, we tested the postsynaptic SO interneurons depolarization in response to a train of alveus stimulations, at 30 Hz. These recordings showed an incremental and persistent depolarization increase with each stimulation in WT (Figures 7E–G). Conversely, in DS *Scn1a*^A1783V/WT^, an increase in the depolarization was evident only following the first stimulus, with subsequent trains resulting in no significant increments (Figures 7E–G). These data are consistent with higher initial release probability in the first stimulation in DS with reduced facilitation in subsequent stimulations. Additionally, we measured the amplitudes of evoked EPSCs and IPSCs and E/I balance. In contrast to the changes observed in CA1 pyramidal neurons, SO interneurons demonstrated no difference between WT and DS *Scn1a*^A1783V/WT^ (Figures 7H–K). Together, lower PPR and synaptic depolarization in DS suggest higher release probability of CA1 pyramidal neurons. These properties may be related to the ability of SO interneurons to preserve their firing within the CA1 microcircuit, despite their reduced excitability.

## Discussion

The prevailing hypothesis for Dravet syndrome neuropathology suggests that its root cause lies with loss of function of Na_V_1.1, leading to dysfunction of multiple types of inhibitory neurons (Mantegazza and Broccoli, 2019). However, spontaneous firing rates of interneurons *in vivo* were not reduced (De Stasi et al., 2016; Tran et al., 2020). Moreover, in brain slices reduced inhibition was not observed in older mice, indicating homeostatic neuronal changes (Favero et al., 2018). Unexpectedly, while characterizing the functional effect in response to the application of Hm1a, we saw that despite hypo-excitability of DS *Scn1a*^A1783V/WT^ and *Scn1a*^R613X/WT^ SO interneurons, their response to Hm1a was preserved. Moreover, using synaptically evoked activity measurements in the hippocampal CA1 microcircuit, we demonstrate DS-related neuronal changes consistent with synaptic alterations in both CA1 pyramidal neurons and SO interneurons. Together, these results suggest neuronal alterations within the CA1 circuit, that may not be limited to loss of function of Na_V_1.1.

### The Interplay Between Stratum-Oriens Interneurons Hypo-Excitability and Na_V_1.1 Function in Dravet Syndrome

Similar to previous studies (Catterall, 2018), we also observed reduced excitability of SO interneurons in DS *Scn1a*^A1783V/WT^, with elevated threshold for AP (Figures 1G, 3F,G) and reduced firing in response to prolonged current injections (Figures 1B,C). Mechanistically, hypo-excitability of inhibitory neurons in DS was assumed to be related to reduced function of Na_V_1.1, because of the Dravet associated *Scn1a* haploinsufficiency. However, if indeed interneurons in DS mice have reduced activity of Na_V_1.1 channels, their response to Hm1a application is expected to be smaller. Surprisingly, WT and DS *Scn1a*^A1783V/WT^ had comparable response to Hm1a, with hyperpolarization of the threshold for APs (Figures 1, 4), and an increase in their firing probability (Figures 4E–G). Moreover, while before the application of Hm1a there was a statistical difference between WT and DS, the effect of Hm1a on the threshold for AP was slightly larger in DS *Scn1a*^A1783V/WT^, causing these differences to dissipate in its presence (Figures 1G,H, 4I,J). Hence, despite the DS associated *Scn1a* loss of function mutation, and the characteristic DS-associated interneurons hypo-excitability, there is a similar functional expression of Hm1a-sensitive sodium channels in both genotypes.

One possible explanation for these unexpected results is that the model used here, harboring the A1783V missense mutation in the *Scn1a* gene, causes Dravet by a different mechanism, that does not involve reduced activity of Na_V_1.1. We deem this possibility less likely because: (i) similar preserved Hm1a effect on firing probability was observed in DS mice harboring the *Scn1a*^R613X^ nonsense mutation, despite reduction in the mRNA levels of *Scn1a* (Figure 5); (ii) the phenotypes of these DS *Scn1a*^A1783V/WT^ mice are the same as those of other models of DS that are based on truncation mutations of the *Scn1a* gene, including the presentation of Dravet associated epileptic and non-epileptic comorbidities, as well as the time course of the disease (Ricobaraza et al., 2019; Fadila et al., 2020; Almog et al., 2021; Miljanovic et al., 2021; Pernici et al., 2021); (iii) the *SCN1A*^*A*1783*V*^ confers loss of function of Na_V_1.1 channels via a right shift of the voltage dependency of activation, as well as left shift of the slow inactivation, resulting in *SCN1A* haploinsufficiency (Layer et al., 2021); (iv) similar to other DS mouse models, *Scn1a*^A1783V/WT^ mice feature reduced excitability of multiple types of inhibitory neurons (Kuo et al., 2019; Almog et al., 2021; Layer et al., 2021); (v) the deficits we observed in the activity of SO interneurons were almost identical to those found in another DS model, harboring a truncation mutation in the *Scn1a* gene (Rubinstein et al., 2015b; Almog et al., 2021).

In accordance with our data, Richards et al. (2018) reported that Hm1a had an effect on interneurons from DS mice harboring the *Scn1a*^R1407X^ truncation mutation, but not in WT mice. These DS mice were shown to express lower levels of Na_V_1.1 (Ogiwara et al., 2007), and are therefore expected to have a smaller response to Hm1a application. Nevertheless, focusing on CA1 stratum radiatum interneurons, Richards et al. (2018) demonstrated that although Hm1a did not affect the firing rates or peak amplitude of AP in WT neurons, it rescued the AP firing deficit and prevented the attenuated spike height accommodation in DS *Scn1a*^R1407X^. Thus, these data from DS *Scn1a*^R1407X^ mice further support our conclusion that despite interneuron hypo-excitability in DS, the response to Hm1a is not reduced. Interestingly, studies that utilized measurements of sodium currents in dissociated neurons or nucleated patches also reported similar sodium current densities in WT and DS neurons at this age group (at seizure onset) (Hedrich et al., 2014; Rubinstein et al., 2015b). Of note, other studies that examined the functional effect of Hm1a did not provide a direct comparison between the genotypes and reported on either DS (Goff and Goldberg, 2019; Mattis et al., 2021) or WT (Chever et al., 2021) interneurons.

Another possible explanation for the lack of differences in the Hm1a impact on WT and DS SO interneurons, is that different Hm1a-sensitive sodium channel(s) repertoire is expressed in each genotype. Moreover, while Hm1a is relatively selective for Na_V_1.1, at 50 nM there might be some modulation of additional Na_V_ channels (Osteen et al., 2016; Richards et al., 2018; Chever et al., 2021). One candidate is Na_V_1.3, which also displays a relatively high affinity to Hm1a (Osteen et al., 2016; Richards et al., 2018), and was suggested to be upregulated in DS mice (Yu et al., 2006). Therefore, it is possible that the upregulation of Na_V_1.3 in DS masks the loss of function of Na_V_1.1, mediating the response to Hm1a. Hippocampal single cells RNA-seq data of juvenile mice (P26–35) showed that the predominate Na_V_ channel in SO interneurons is *Scn1a*, with an expression of ∼180 Fragments Per Kilobase Million (FPKM), in contrast to *Scn2a* (∼80 FPKM), *Scn3a* (∼8 FPKM), and *Scn8a* (∼40 FPKM) (Cembrowski et al., 2016). Thus, with these expression levels, Na_V_1.3 should be significantly upregulated to mediate comparable responses to Hm1a. However, while immunohistological analysis indicated a dramatic increase in Na_V_1.3 expression (Yu et al., 2006), RNA-seq and proteomic analysis of hippocampi from DS mice did not show substantial upregulation of any Na_V_ subtype, including Na_V_1.3 (Hawkins et al., 2019; Miljanovic et al., 2021).

In addition to its effect on voltage gated sodium channels, Hm1a can also inhibit voltage gated potassium channels. However, the affinity to potassium channels was reported to be lower, with ∼20% inhibition of K_V_2.1 and K_V_2.2 at 100 nM (Escoubas et al., 2002), and ∼40% inhibition of K_V_4 channels (Escoubas et al., 2002) and ∼10% inhibition of K_V_11.1 (hERG) (Richards et al., 2018) by 300 nM. Thus, at 50 nM, the Hm1a concentration used here, this toxin is expected to be relatively selective for sodium channels. Nevertheless, as these specificity and affinity studies were performed in expression systems, and these parameters may differ in brain slices, we cannot exclude the possible contribution of weak inhibition of potassium channels to the observed preservation of the Hm1a response in DS interneurons.

Furthermore, as our results are based on stimulations at low frequency (1 Hz), it remains to be determined if stimulation at higher frequencies can expose additional neuronal alterations in DS that can be directly correlated with loss of function of Na_V_1.1.

Together, we demonstrate that the functional effect of Hm1a was preserved in two DS models, harboring a missense or a nonsense mutation in the *Scn1a* gene, despite reduced excitability of SO interneurons. Based on these observations, we propose that during epileptogenesis, genetic *Scn1a* mutations, that cause reduced activity of Na_V_1.1, trigger additional complex neuronal changes that eventually lead to the apparent and unexpected, preserved functional response to Hm1a. Indeed, DS-related dysregulations of voltage-gated potassium and calcium channels were reported before (Goff and Goldberg, 2019; Ritter-Makinson et al., 2019; Miljanovic et al., 2021), indicating the involvement of multiple ion channels in Dravet pathophysiology.

### CA1 Pyramidal Neurons of Dravet Syndrome *Scn1a*^A1783V/WT^ Mice Demonstrate Reduced Effect of Hm1a

The role of excitatory neurons in DS pathophysiology is debated. While the firing of these neurons in brain slices is mostly unaltered in DS (Yu et al., 2006; Tai et al., 2014; Rubinstein et al., 2015b; De Stasi et al., 2016), others have reported changes in firing or dendritic arborization (Mistry et al., 2014; Tsai et al., 2015; Salgueiro-Pereira et al., 2019; Almog et al., 2021). Similar to previous reports (Richards et al., 2018; Chever et al., 2021), our data also demonstrate that, when firing was evoked by direct injection of depolarizing currents through the patch pipette, Hm1a application did not affect the firing rates of CA1 excitatory neurons (Figures 2A–D). In contrast, diverse effects of Hm1a were observed in response to synaptic stimulation, in which excitatory inputs are received and processed by the dendrites. Interestingly, when the firing was evoked by stimulation of the SC, Hm1a application increased the firing probability of CA1 pyramidal neurons (Figures 2H–K), reduced the threshold voltage for eAP (Figures 2L,M) and augmented the CA1 fEPSP in WT neurons (Figures 4B–D). These Hm1a induced changes indicate the contribution of Na_V_1.1 channels to the amplification of synaptic inputs in CA1 pyramidal neurons (Stuart and Sakmann, 1995; Fricker and Miles, 2000; González-Burgos and Barrionuevo, 2001). Contrariwise, these Hm1a effects were absent in DS mice (Figures 2H–M, 4B–D), indicating reduced functional expression of dendritic Hm1a-sensitive sodium channels in DS mice.

The RNA-seq data of WT juvenile mice indicated abundant expression of Na_V_1.2 and Na_V_1.6 in these neurons (*Scn2a*: ∼120 FPKM; *Scn8a*: ∼90 FPKM), low expression of Na_V_1.3 (*Scn3a*: ∼11 FPKM), and intermediate expression of Na_V_1.1 (*Scn1a*: ∼40 FPKM, 4.5-fold lower compared to their expression in SO interneurons) (Cembrowski et al., 2016). While Na_V_1.6 and Na_V_1.2 are the predominant sodium channels found in the AIS (Hu et al., 2009; Lorincz and Nusser, 2010; Katz et al., 2018), and Na_V_1.2 are the primary dendritic sodium channels (Spratt et al., 2021), the spatial expression of Na_V_1.1 in these cells remains poorly defined, with evidence supporting somato-dendritic expression (Westenbroek et al., 1989; Yu et al., 2006). Since Hm1a shows some activity also toward Na_V_1.2 channels (Chever et al., 2021), and due to the high dendritic expression of these channels in CA1 pyramidal neurons (Cembrowski et al., 2016), we cannot exclude the possibility that the reduced effect of Hm1a in DS *Scn1a*^A1783V/WT^ is governed by alterations in the functional expression of Na_V_1.1 and Na_V_1.2 (Figures 2, 4A–D). Nevertheless, while reduced activity of Na_V_1.1 can be directly linked to the DS-associated *Scn1a*^*A*1783*V*^ mutation, the molecular mechanisms that may lead to DS-related reduction in the expression levels of Na_V_1.2 remain to be determined. Together, these data indicate that excitatory neurons of DS *Scn1a*^A1783V/WT^ mice display divergent expression of Hm1a-sensitive channels, highlighting alterations in Na_V_ functions in these cells.

### Preserved Firing Probability of Stratum-Oriens Interneurons Within the CA1 Microcircuit

Dravet syndrome associated seizures and age-dependent homeostatic changes, extending beyond the loss of function of Na_V_1.1, were reported before. These included developmental changes and correction of the hypo-excitability of cortical inhibitory neurons (Favero et al., 2018), age-dependent changes in the activity of hippocampal inhibitory and excitatory neurons (Almog et al., 2021), seizure-induced hyper-excitability of dentate gyrus granule cells (Salgueiro-Pereira et al., 2019), as well as maturation related changes in field potential paired-pulse facilitation (Liautard et al., 2013).

Our data add additional findings that indicate reduced synaptic strength between CA3 and CA1 (Figures 3C–E,H). Specifically, we observed that increased fiber volley amplitudes were needed to produce comparable fEPSP (Figures 3C–E,H). Moreover, we show reduced synaptic conductances in CA1 pyramidal neurons from DS mice (Figures 6H–J). While Na_V_1.1 haploinsufficiency may contribute to reduced boosting of incoming EPSPs (Stuart and Sakmann, 1995; Fricker and Miles, 2000; González-Burgos and Barrionuevo, 2001; Carter et al., 2012), reduced excitatory and inhibitory conductances are probably related to reduced connectivity or lower expression of synaptic receptors. Interestingly, a recent comparative proteomic analysis of hippocampal cells from DS mice demonstrated wide expressional changes, with variations in multiple types of proteins involved in excitatory and inhibitory synaptic transmissions, including lower levels of glutamate and GABA receptors (Miljanovic et al., 2021). Moreover, reduced synaptic conductances were reported in multiple mouse models of autism, and were suggested to be related to homeostatic synaptic changes (Antoine et al., 2019).

Based on these deficits in the CA3-CA1 synapse and SO excitability, we hypothesized an additive reduction in the firing probability of SO interneurons in response to SC stimulation. Nevertheless, surprisingly, the firing probability of SO interneurons was unaltered in DS *Scn1a*^A1783V/WT^, along a range of CA3 stimulation intensities (Figures 3I,J). This preserved firing may be related to synaptic changes that we observed in the CA1-SO synapse, including lower PPR, indicating higher initial release probability from CA1 terminals, as well as the lack of change in the synaptic conductances onto the SO interneurons (Figure 7).

Despite these unexpected data, reduced inhibition in DS was demonstrated to be related to the pathophysiology, and was also demonstrated here (Figures 1, 3–6). Indeed, selective genetic perturbation of the *Scn1a* gene in interneurons was sufficient to recapitulate key pathophysiological features of Dravet in mice (Cheah et al., 2012; Ogiwara et al., 2013; Rubinstein et al., 2015a; Tatsukawa et al., 2018; Kuo et al., 2019). Moreover, the overall frequency of inhibitory inputs onto CA1 neurons was shown to be reduced in DS (Han et al., 2012; Liautard et al., 2013; Almog et al., 2021). Furthermore, our data also demonstrate increased E/I balance within the CA1 microcircuit (Figure 6K). Nevertheless, rather than reduced firing probability, disinhibition may be related to a reduction in synaptic inhibitory connections onto pyramidal neurons, reduced number of interneurons (Dyment et al., 2020), reduced expression of GABA_A_R (Miljanovic et al., 2021), or alterations in the reversal potential of GABA_A_R (Yuan et al., 2019). Moreover, it is possible that SO interneurons can preserve normal firing when stimulated at 1 Hz but would fail at high frequencies. Indeed, several studies demonstrated that DS interneurons can preserve firing at low frequencies (Yu et al., 2006; Hedrich et al., 2014; Favero et al., 2018; Lemaire et al., 2021). Furthermore, while we focused on the activity of SO interneurons (Figure 3), reduced firing of parvalbumin (PV) positive interneurons governs the observed deficits in the E/I balance. Over 80% of SO interneurons are somatostatin (SST) positive O-LM cells (Tricoire et al., 2011; Winterer et al., 2019; Almog et al., 2021). Studies of conditional mice with selective deletion of the *Scn1a* gene in PV or SST interneurons showed that loss of function of *Scn1a* in PV neurons results in more severe phenotypes compared to selective deletion in SST neurons (Rubinstein et al., 2015a; Tatsukawa et al., 2018). However, deletion in both types of interneurons had a more profound effect (Rubinstein et al., 2015a). Thus, while DS epilepsy involves inhibitory neuron dysfunctions, the neuronal mechanism may be more complex than reduced firing probability.

## Conclusion

In conclusion, our data reveal synaptic and excitability alterations in both CA1 excitatory neurons and CA1 SO interneurons in DS mice, supporting global homeostatic changes within the CA1 microcircuit that may partially compensate for the DS-related interneurons hypo-excitability.

## Data Availability Statement

The raw data supporting the conclusions of this article will be made available by the authors, without undue reservation.

## Ethics Statement

The animal study was reviewed and approved by the Animal Care and Use Committee (IACUC) of Tel Aviv University.

## Author Contributions

YA and MR conceived and designed the experiments, analyzed the data, and wrote the manuscript. YA, AM, and MB performed the experiments. All authors contributed to the article and approved the submitted version.

## Conflict of Interest

The authors declare that the research was conducted in the absence of any commercial or financial relationships that could be construed as a potential conflict of interest.

## Publisher’s Note

All claims expressed in this article are solely those of the authors and do not necessarily represent those of their affiliated organizations, or those of the publisher, the editors and the reviewers. Any product that may be evaluated in this article, or claim that may be made by its manufacturer, is not guaranteed or endorsed by the publisher.
