# Supplementary material for: Functional Investigation of a Neuronal Microcircuit in the CA1 Area of the Hippocampus Reveals Synaptic Dysfunction in Dravet Syndrome Mice
Source: Front Mol Neurosci. 2022 Mar 16;15:823640. doi: 10.3389/fnmol.2022.823640 (PMC8966673; doi:10.3389/fnmol.2022.823640)
Supplement: Supplementary file 1 [file Data_Sheet_1.PDF]

Functional dissection of the CA1 microcircuit using a specific Na$_V$1.1 activator reveals synaptic dysfunction in Dravet syndrome mice

Yael Almog[1,2], Anat Mavashov[1,3], Marina Brusel[1], Moran Rubinstein[1,2,3]

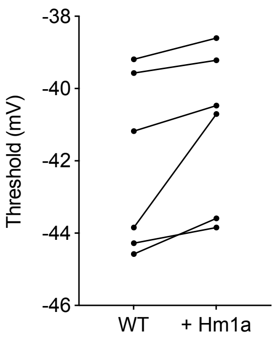

Supplementary Fig. 1. 10 nM Hm1a had no statistical effect on the threshold for AP in WT SO interneurons (n=6). These data followed a normal distribution. Paired t-test p = 0.062
